# Total HLA Class I Antigen Loss with the Downregulation of Antigen-Processing Machinery Components in Two Newly Established Sarcomatoid Hepatocellular Carcinoma Cell Lines

**DOI:** 10.1155/2018/8363265

**Published:** 2018-12-16

**Authors:** Wei-Yi Lei, Shih-Chieh Hsiung, Shao-Hsuan Wen, Chin-Hsuan Hsieh, Chien-Lin Chen, Christopher Glenn Wallace, Chien-Chung Chang, Shuen-Kuei Liao

**Affiliations:** ^1^Institute of Medical Sciences, Tzu Chi University, Hualien, Taiwan; ^2^College of Medicine, Tzu Chi University, Hualien, Taiwan; ^3^Department of Medicine, Hualien Tzu Chi Hospital, Buddhist Tzu Chi Medical Foundation, Hualien, Taiwan; ^4^Division of Urology, Department of Surgery, Chang Gung Memorial Hospital, Taoyuan, Taiwan; ^5^Institute of Molecular and Cellular Biology, National Tsing Hua University, Hsinchu, Taiwan; ^6^Department of Plastic and Reconstructive Surgery, Chang Gung Memorial Hospital, Taoyuan, Taiwan; ^7^Department of Life Science, National Tsing Hua University, Hsinchu, Taiwan; ^8^The Ph.D. Program for Cancer Biology and Drug Discovery, Taipei Medical University, Taipei City, Taiwan; ^9^Vectorite Biomedica/U-Well Biopharma Inc., New Taipei City, Taiwan

## Abstract

Limited information is currently available concerning HLA class I antigen abnormalities in sarcomatoid hepatocellular carcinoma (sHCC). Here, we have analyzed the growth characteristics and HLA class I antigen status of four sHCC cell lines (sHCC29, sHCC63, sHCC74, and SAR-HCV); the first three were newly established in this study. Among the four, sHCC29 showed the highest growth rate *in vitro* and tumorigenicity in NOD-SCID mice. Unlike sHCC74 and SAR-HCV, both sHCC29 and sHCC63 had no detectable surface HLA class I antigen expression, alongside undetected intracellular *β*_2_-microglobulin (*β*_2_m) and marked HLA class I heavy chain and selective antigen-processing machinery (APM) component downregulation. The loss of *β*_2_m in sHCC29 and sHCC63 was caused by a >49 kb deletion across the *B2M* locus, while their downregulation of APM components was transcriptional, reversible by IFN-*γ* only in several components. *β*_2_m was also undetected in the primary HCC lesions of the patients involved, indicating its *in vivo* relevance. We report for the first time HLA class I antigen loss with underlying *B2M* gene deficiency and APM defects in 50% (2 of 4) of the sHCC cell lines tested. These findings may have implications for a proper design of T cell immunotherapy for the treatment of sHCC patients.

## 1. Introduction

Hepatocellular carcinoma (HCC) is the 6th most common solid tumor and the third leading cause of cancer-related death worldwide [1, 2]. Although the role of hepatitis B virus (HBV) and HCV in hepatocarcinogenesis has been well documented, its precise mechanism remains largely unknown, especially for HCV-associated hepatocarcinogenesis. It has been reported that some liver tumors, including HCC, cholangiocarcinoma, and hepatocholangiocarcinoma, may undergo sarcomatous change [3], a phenomenon closely associated with epithelial-mesenchymal transition (EMT) and neoplastic progression [4, 5]. Notably, the primary liver sarcomatous carcinomas have been found to arise with or without cirrhosis pathology [6]. Although sarcomatous liver tumors are rare with only about 50 cases reported thus far in the English medical literature [7–10], these sarcomatoid carcinomas are highly aggressive, characterized by a fast clinical course with a poor prognosis [10, 11]. No effective therapy is currently available for the treatment of this rare tumor subtype.

It has been known for some time that malignant cells can have impaired HLA class I antigen expression during the course of tumor progression [12–15]. HLA class I antigen downregulation or loss often occurs in tumor cells when the primary tumor breaks the basal membrane, invades the surrounding tissues, and/or begins to metastasize to regional lymph nodes or to distant organs with the latter occurring at higher frequencies [16, 17]. Since HLA class I antigens present tumor antigen-derived peptides to the host cytotoxic T lymphocytes (CTLs), altered or deficient HLA class I antigen expression by malignant cells constitutes an effective mechanism to escape HLA class I-restricted T cell antitumor surveillance. The clinical relevance of HLA class I antigen downregulation or loss in tumors has been indicated by its association with poor prognosis of several malignant diseases, including melanoma, breast cancer, and clear cell renal cell carcinoma [17]. In a therapeutic setting, the effector function of T cells could be dampened by the HLA class I antigen abnormalities in tumors, and this may pose an obstacle to therapeutic success. This possibility may explain the outcome of the recent T cell immune checkpoint blockade therapy of melanoma [18] and NSCLC [19], which is effective but with only 20–25% of response rate.

The HLA class I antigen status in tumor cells may represent a key variable for the efficacy of therapy that relies on CTLs to eliminate the tumor cells [20]. Primary HCCs have been reported to have sufficient levels of HLA class I antigens expressed [21, 22], yet currently, no information is available regarding the expression status of these molecules in the sarcomatoid subtype of HCC (sHCC) and its association with prognosis of the disease. Therefore, we herein established three sHCC cell lines derived from the surgically removed liver tumors of three patients with apparent sarcomatoid changes in the lesion; one is HCV-related while the other two are HBV-related. Together with a previously established sHCC cell line known as SAR-HCV [23], we have analyzed their HLA class I antigen expression and found that two of the four cell lines harbored a large deletion in the *β_2_m* gene, associated with downregulation of several components in the HLA class I antigen presentation pathway.

## 2. Materials and Methods

### 2.1. The Patients

The clinical histories of patients from whom the three newly established sHCC cell lines originated are as follows.

#### 2.1.1. Case 1 (sHCC29)

A 58-year-old woman with history of liver cancer originating from chronic hepatitis B was admitted to Taipei Tzu Chi Hospital, Taiwan, in August 2010, with chief complaints of yellowish discoloration of the skin and tea-color urine for one week. She has received transcatheter arterial chemoembolisation (TACE) three months prior to admission to this hospital. Laboratory tests showed elevated serum total bilirubin (5.1 mg/dL), alpha-fetoprotein (182.7 ng/mL), and carbohydrate antigen 19-9 (70.5 U/mL) levels. The carcinoembryonic antigen (CEA) serum level was within the upper limit of normal. Computed tomography revealed a hypervascular mass in the liver hilum measuring 4 × 5 cm. The patient underwent left lobectomy, and pathology showed proliferation of spindle-shaped hepatocellular carcinoma cells. The patient has survived for 5 additional years without tumor recurrence as of this writing.

#### 2.1.2. Case 2 (sHCC63)

A 39-year-old man with a history of HBV-related cirrhosis was referred to Hualien Tzu Chi General Hospital, Taiwan, in May 2011, diagnosed as having liver cancer without any previous treatment for HCC. The alpha-fetoprotein serum level was 123.5 ng/mL. Both the carbohydrate antigen 19-9 and CEA serum levels were within the normal range. The combination of computed tomography with hepatic arteriography and arterial portography (CTHA/CTAP) showed a huge hypervascular tumor in the right lobe of the liver. The patient underwent liver resection. Histological examination revealed spindle-shaped sarcomatoid carcinoma cells with unclear trabecular and pseudoglandular structures. However, the tumor relapsed in the residual liver 5 months after surgery, and despite TACE therapy, the patient died one year later.

#### 2.1.3. Case 3 (sHCC74)

A 72-year-old woman was admitted to Hualien Tzu Chi General Hospital, Taiwan, and diagnosed as having liver cancer originating from chronic hepatitis C. The alpha-fetoprotein, carbohydrate antigen 19-9, and CEA serum levels were all within the upper limit of normal. Computed tomography showed a hypervascular tumor in the caudate lobe of the liver measuring 5 × 6 cm. She underwent segmentectomy, and pathology revealed spindle-shaped sarcomatoid carcinoma cells. One year after surgery, the tumors relapsed in the residual liver. The patient began with TACE therapy. Unfortunately, she died two years later.

### 2.2. Establishment of sHCC Cell Lines

Three sHCC cell lines were established from HCC tissues during surgery performed at Hualien Tzu Chi Hospital with patients' consent. The study has been approved by the Research Ethics Committee in Tzu Chi General Hospital (IRB 101–62). The three sHCC cell lines (sHCC29, sHCC63, and sHCC74) were established according to standard procedures. Essentially, the minced HCC tissues were pretreated with collagenase, washed, and cultivated on the mitomycin C-treated NIH/3T3 feeder layer for 4 to 6 passages to select the HCC cell lines. Homogenous HCC cell populations were obtained, and sustained proliferation was observed over 30 passages in culture *in vitro*. Cultured cell lines between 18 and 24 passages were used in the subsequent studies.

The fourth sHCC cell line known as SAR-HCV cell line was previously established from a malignant liver lesion of a 68-year-old male patient infected with HCV. Its initial characterization has been reported previously [23, 24].

### 2.3. Xenotransplantation of Four Established sHCC Cell Lines into Severe Combined Immunodeficiency Mice

Six-week-old female Non-Obese Diabetic (genetic background)/Severe Combined Immunodeficiency (SCID) mice were purchased from BioLASCO, Taiwan Co. Ltd., Taipei, Taiwan. Tumorigenicity assays of the three cell lines (sHCC29, sHCC63, and sHCC74) were carried out in such mice in this study (*n* = 5/group). The experimental protocol was approved by the Animal Ethics Committee, Chang Gung University, Taoyuan, Taiwan. Single cell suspensions of the three cell lines were prepared from monolayer cultures by light trypsinization and washing in phosphate-buffered saline (PBS). Cells were then counted, and 10^7^ cells/0.1 mL PBS were inoculated into the flank of each mouse. The animals were examined every 2 days to monitor the growth of tumors. The volume of palpable tumor nodules was calculated according to the formula volume(mm^3^) = 0.4 × *a* × *b* ^2^, where (*a*) is the major diameter, and (*b*) is the minor diameter perpendicular to the major one.

### 2.4. IFN-*γ*

Recombinant human IFN-*γ* was purchased from R&D Systems, Inc. (Minneapolis, MN).

### 2.5. Monoclonal Antibodies (mAb) and Polyclonal Antibodies

The mAb W6/32, which recognizes *β*_2_m-associated HLA-A, HLA-B, HLA-C, HLA-E, and HLA-G heavy chains [25, 26], was purchased from Thermo Fisher Scientific, Fremont, CA. The mAb TP25.99, which recognizes *β*_2_m-free and *β*_2_m-assciated HLA-A, HLA-B, and HLA-C heavy chains [27], the mAb B1.23.1, which recognizes *β*_2_m-associated HLA-B, HLA-C heavy chains [28], the mAb HC10, which recognizes a determinant expressed on *β*_2_m-free HLA-A10, HLA-A28, HLA-A29, HLA-A30, HLA-A31, HLA-A32, and HLA-A33 heavy chains and on all HLA-B and HLA-C heavy chains [29, 30], the mAb HCA2, which recognizes a determinant expressed on *β*_2_m-free HLA-A (except HLA-A24), HLA-B7301 and HLA-G heavy chains [31], the *β*_2_m-specific mAb L368 [32], the LMP-2-specific mAb SY-1 [33], the LMP7-specific mAb HB2 [34], the TAP1-specific mAb NOB1 [35], the TAP2-specific mAb NOB2, and the HLA-DR, HLA-DP, HLA-DQ-specific mAb LGII-612.14 [34] were previously produced and characterized. Fluorescein-isothiocyanate conjugated goat anti-mouse IgG antibodies were purchased from DAKO (Glostrup, Denmark).

### 2.6. Cytofluorometric Analysis

Cell surface and cytoplasmic staining with mAbs were conducted as described previously [34]. Purified normal mouse serum IgG or an isotype-matched mouse myeloma IgG was used as specific negative controls. Stained cells were analyzed by using a BD FACSVerse™ flow cytometer (BD Biosciences, San Jose, CA). Results were expressed as % positively stained cells and as mean fluorescence intensity (MFI).

### 2.7. Reverse-Transcriptase-Polymerase Chain Reaction (RT-PCR) and Genomic PCR

Total RNA was isolated utilizing the TRIZOL reagent (Invitrogen, Carlsbad, CA) following the manufacturer's instructions. RT-PCR was performed with the primers for *β_2_m*, *LMP2*, *LMP7*, *TAP1*, *TAP2*, and glyceraldehyde-3-phosphate dehydrogenase (*GAPDH*) as a loading control (Table 1). The following PCR conditions were used: 40 cycles at 95°C for 1 min, 60°C for 1.5 min, and 72°C for 2 min with a 10 min extension after the last cycle. Genomic DNA was isolated from sHCC cells utilizing the mammalian genomic DNA extraction mini-prep kit (Sigma, Dorset, England) according to the manufacturer's instructions. PCR was initially carried out using *B2M* gene-specific primers, forward 744F and reverse 468R, to amplify the promoter-to-intron 1 region. For determining the length of deletion in the gene, a panel of additional primers encompassing the *B2M* gene locus at 15q15 was designed based on NCBI GenBank Accession AC018901 (Supplementary Table 1) and used in PCR. The PCR products were fractionated on a 1% agarose gel (Roche, Indianapolis, IN) and visualized by ethidium bromide staining. For bands from RT-PCR, the density of bands was read by a densitometer, ChemiSmart 3000, equipped with the Bioprofil 1D++ software (Viber Lournat, Marne-la-Vallee, France). The relative level of transcript in each experimental group was estimated after normalization with the density of the resulting *GAPDH* band of the same experimental group.

### 2.8. Immunohistochemistry

Three-micron-thick affined formalin-fixed paraffin-embedded tumor blocks obtained from patients were processed in the Pathology Department, Tzu Chi General Hospital in Hualien, Taiwan. Prior to immunostaining, the deparaffinized slides were subjected to an antigen-retrieval process by dipping the slides in a breaker containing 0.01 M sodium citrate (pH 6.0) in a boiling state on a hot plate. Following a 20 min incubation, the breaker was removed from the hot plate and cooled down at room temperature for 20 min. Slides were washed once in PBS and stained with mAbs using avidin-biotin-peroxidase complex (ABC) method with the UltraVision Quanto Detection System HRP DAB kit (Lab Vision Corporation, Fremont, CA) according to the manufacturer's instructions.

## 3. Results

### 3.1. *In Vitro* Morphological and Growth Characteristics of sHCC Cell Lines

**Figure 1:**
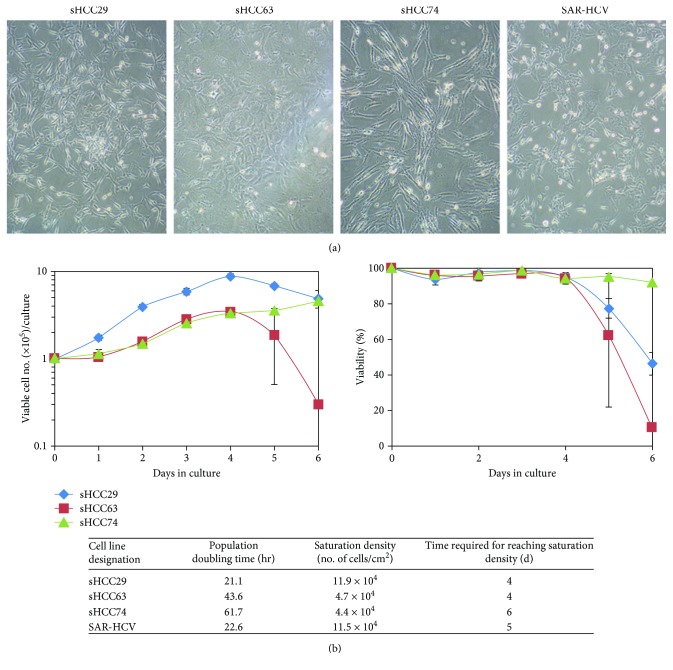
*In vitro* live monolayer cell morphology and growth curves of the four sHCC cell lines. (a) Fibroblastic-like morphology is shown for each of the sHCC cell lines, sHCC29, sHCC63, sHCC74, and SAR-HCV, as they grow as monolayers. (b) Growth curve patterns of the first three sHCC are illustrated. The growth curve of SAR-HCV is not shown as it has been reported previously [23]. The population doubling time and time required for reaching saturation density in culture for each cell line based on the growth curve are listed in the attached table.

Typical fibroblastic morphology in a rather disorganized orientation was exhibited by each of the four sHCC cell lines when they were cultured *in vitro* as monolayers, although some subtle differences were noted among them (Figure 1(a)). For example, sHCC63 and sHCC74 showed longer cell bodies as compared with those of sHCC29 and SAR-HCV. In terms of growth characteristics, growth curves of the four cell lines are shown with somewhat different patterns (Figure 1(b)). sHCC29 and SAR-HCV had much shorter population doubling time (21.1 and 22.6 h *vs*. 43.6 and 61.7 h) and much higher saturation density (11.9 × 10^4^ and 11.5 × 10^4^ cells/cm^2^ *vs*. 4.7 × 10^4^ and 4.4 × 10^4^ cells/cm^2^), when compared with other two sHCC cell lines, sHCC63 and sHCC74 (table under Figure 1(b)). No viral DNA (HBV and/or HCV) was detected in the culture supernatants or cell extracts from any of the four sHCC cell lines by real-time PCR (data not shown).

### 3.2. Tumorigenicity in Immunodeficient Mice

**Figure 2:**
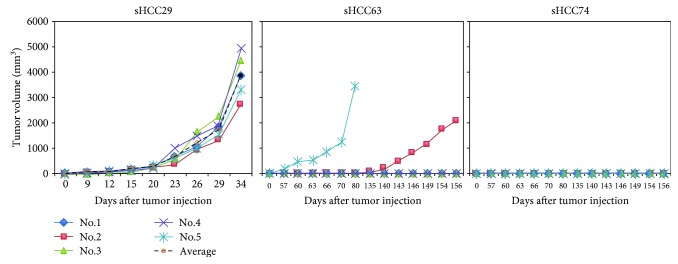
*In vivo* tumorigenicity of the three sHCC cell lines, sHCC29, sHCC63, and sHCC74, in NOD/SCID mice. Results on SAR-HCV are not shown, as they have been reported elsewhere [24].

Xenotransplantation abilities in NOD/SCID mice were tested by subcutaneous injection of 10^7^ monodispersed cells harvested from sHCC29, sHCC63, and sHCC74 per mouse (Figure 2). Results showed that sHCC29 cells gave rise to solid tumors at the injection sites in 5/5 mice injected with a similar latent period (15 days); sHCC63 resulted in only 2/5 animals injected with quite different latent periods of 50 *vs.* 135 days, while injection of sHCC74 failed to develop any tumors during 156 days of observation following tumor injection at day 0. As for SAR-HCV, 4 out of 4 mice injected developed solid tumors at the injection sites but with a much longer average latent period of about 4 months [24], as compared with other 3 sHCC cell lines.

### 3.3. Lack of Surface HLA Class I Antigen and *β*_2_m Expression and Downregulation of Several Antigen-Processing Machinery (APM) Components by the sHCC29 and sHCC63 Cell Lines

**Figure 3:**
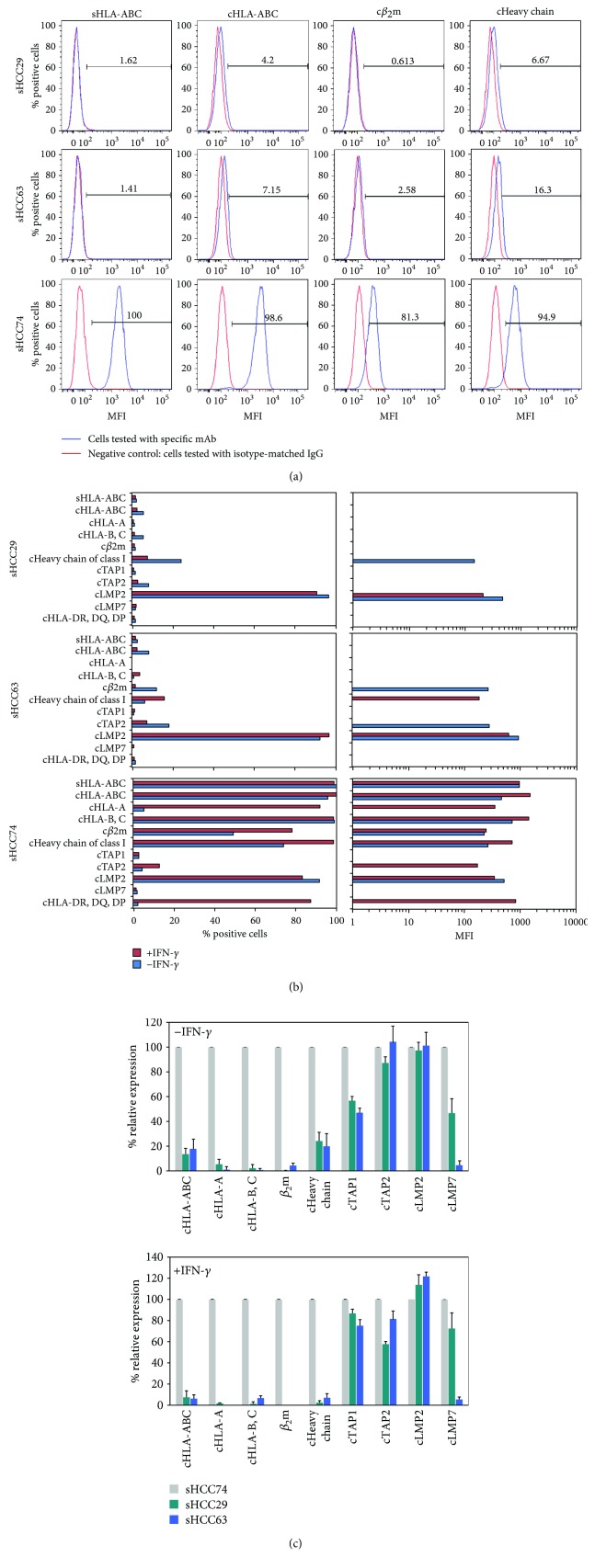
Differential expression of surface HLA class I antigens and various cytoplasmic antigens including *β*_2_-microgobulin (*β*_2_m), heavy chains, and selected antigen-processing machinery (APM) components in three newly established sHCC cell lines. (a) Surface HLA class I (sHLA-ABC) using mAb W6/32 and cytoplasmic HLA class I (cHLA-ABC) using mAb TP25.99, *β*_2_m, and heavy chains (cHC) of the three sHCC cell lines were determined cytofluormetrically, and the results of a representative experiment were presented by histograms. (b) Cytoplasmic expression of additional HLA-A, HLA-B, -C allospecificities and APM components TAP1, TAP2, LMP2, and LMP7 were determined cytofluormetrically. The data are representative of three independent experiments. Symbols “s” and “c” at the prefix of indicated antigen stand for surface and cytoplasmic antigens, respectively. The results of cytofluorometric analysis are expressed as % positive cells on the left frame and mean fluorescence intensity (MFI) on the right frame. (c) Percent expression relative to sHCC74 of HLA class I allospecificities, heavy chains, *β*_2_m, and selected APM components in sHCC29 and sHCC63 are shown. Results are expressed as percent MFI ± SD of three independent experiments. TAP: transporter associated with antigen processing; LMP: low-molecular-weight protein.

Among the four sHCC cell lines tested, only the cell lines sHCC29 and sHCC63 had no detectable surface HLA class I antigen expression as analyzed by cytofluorometry (Figures 3(a) and 3(b) in histogram and bar-type of expression, respectively). *β*_2_m expression in sHCC29 and sHCC63 was undetectable and failed to be restored by incubation of cells with IFN-*γ* (300 U/mL, 48 h) *in vitro* (Figure 3(b)). Of note is that the expressions of surface HLA class I tested using both mAb W6/32 and mAb TP25.99 were identical, although the results obtained with the latter were not shown. In addition, the HLA class I heavy chain expression was markedly downregulated only in the two *β*_2_m-negative cell lines. Among the analyzed APM components, TAP1 was expressed at a low frequency in the two *β*_2_m-negative cell lines, and while LMP2 was abundantly expressed in all of three cell lines, LMP7 expression was detectable only at a very low percentage of sHCC63 cells (Figure 3(c)). These findings indicate that in sHCC29 and sHCC63 cells, *β*_2_m loss was not the only defect underlying HLA class I antigen loss, since it was associated with additional APM defects. Notably, upregulation by IFN-*γ* (300 U/mL, 48 h) of HLA-A, -B, -C heavy chain expression was detectable only in sHCC63 cells.

### 3.4. Detection of a Large (>49 kb) Deletion of the *β_2_m* Gene in the sHCC29 and sHCC63 Cell Lines

**Figure 4:**
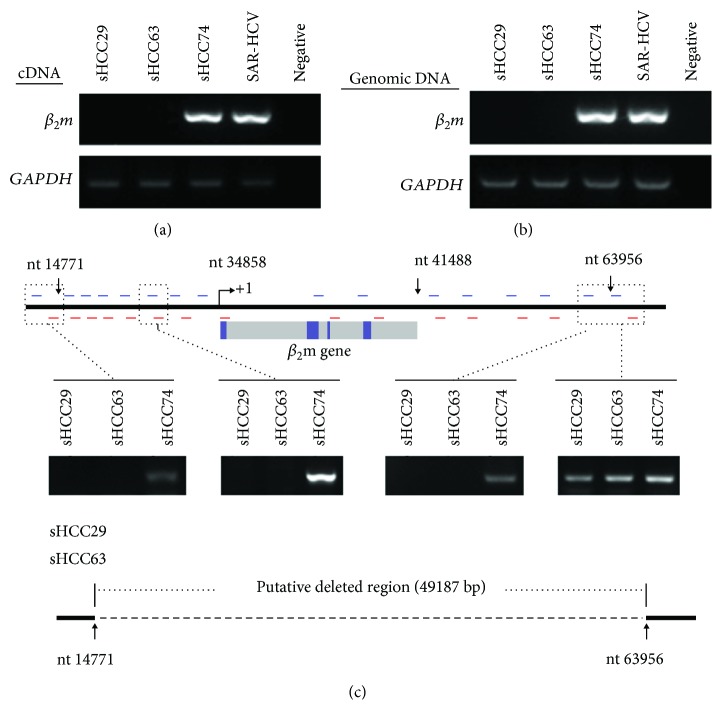
No detection of *β_2_m* cDNA (a) and the *B2M* promoter-to-intron 1 region (b) in both sHCC29 and sHCC63 cells by RT-PCR and genomic PCR, respectively, as compared to sHCC74 and SAR-HCV cells. Schema illustrates the putative deleted region of the *B2M* gene in the two HLA class I-negative sHCC29 and sHCC63 cell lines (c). MFI: mean fluorescence intensity. See the text for detailed explanation.

In addition to the failure of RT-PCR to detect *β_2_m* cDNA (Figure 4(a)), genomic PCR also failed to detect the *B2M* promoter-to-intron 1 region (Figure 4(b)) in both sHCC29 and sHCC63 cells, compared to sHCC74 and SAR-HCV cells. These findings indicate a deletion of the *B2M* gene in the two *β*_2_m-negative cell lines. To determine the length of this deletion, PCR analysis of their *B2M* locus utilizing a panel of 16 primer pairs encompassing the entire 15q15 region was performed. The results show that in both cell lines the deletion was at least 49 kb in length, with a 3′ breakpoint mapped approximately 22 kb (22,468 bp, nt 63,956) downstream of the *B2M* gene and a not-yet-identified 5′ breakpoint extending at least 27 kb (nt 14,771) upstream. These findings indicate that *β*_2_m loss in both sHCC29 and sHCC63 cells was caused by a large deletion in the *B2M* gene that completely eliminated its coding sequence (Figure 4(c)).

### 3.5. Effect of IFN-*γ* on the *β_2_m*, *LMP2*, *LMP7*, *TAP1*, and *TAP2* Transcripts in the Three sHCC Cell Lines

**Figure 5:**
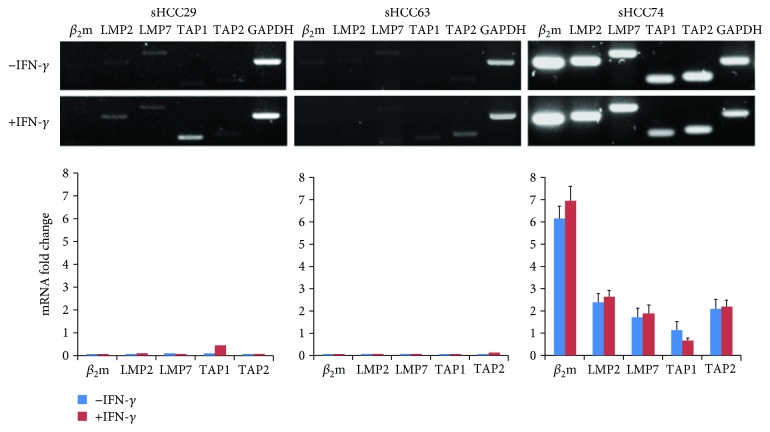
Differential expression of *β_2_m*, *LMP2*, *LMP7*, *TAP1*, and *TAP2* mRNA in the three newly established sHCC cell lines cultured in the presence or absence of IFN-*γ* (300 U/mL, 48 h). Upper panel: agarose gel stained with ethidium bromide shows the electrophoresis of end point RT-PCR products for the tested mRNAs of the three sHCC cell lines. Lower panel: quantification of RT-PCR results from three independent experiments where relative mRNA expression of each gene was normalized to glyceraldehydes 3-phosphate dehydrogenase (*GAPDH*) and expressed as mean fold-changes ± SD. LMP: low-molecular-weight protein; TAP: transporter associated with antigen processing.

Earlier, we have detected downregulation of several APM components at the protein level in the sHCC29 and sHCC63 cells. Here, we tested whether these abnormalities reflected downregulation at the transcriptional level reversible by IFN-*γ*, although this cytokine failed to do so at the protein level for most of the tested components (Figure 3).  Figure 5 shows that the mRNA transcript levels of the tested APM components *LMP2*, *LMP7*, *TAP1*, and *TAP*2 were markedly lower in the sHCC29 and sHCC63 cells than those in the sHCC74 cells. Following treatment with IFN-*γ* (300 U/mL, 48 h), only *TAP1* mRNA expression in sHCC29 and only *TAP2* mRNA expression in sHCC63 cells were upregulated. Expression of the tested APM component mRNA in sHCC74 cells was abundant, and the level of expression was unaffected by IFN-*γ* (300 U/mL, 48 h).

### 3.6. Phenotypic Expression of CD44 and CD24 on the Four sHCC Cell Lines

**Figure 6:**
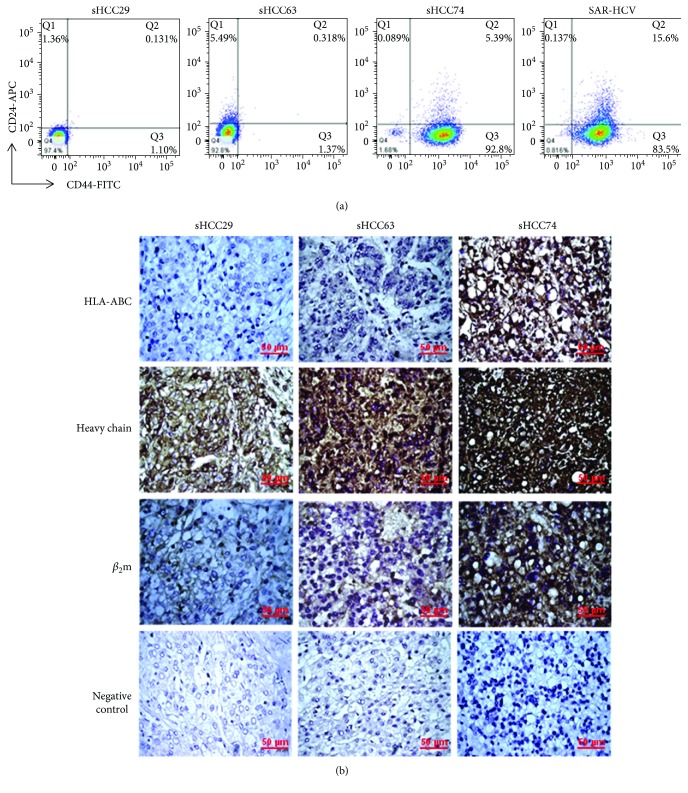
Two-color cytofluorometric analysis of the sHCC29, sHCC63, sHCC74, and SAR-HCV cell lines using mAbs to surface markers CD24 and CD44 conjugated with allophycoyanin (APC) and fluorescein isothioyanate (FITC), respectively (a). Immunohistochemical staining patterns of the tumor lesions from which the sHCC29, sHCC63, and sHCC74 cell lines were established using mAbs to HLA-ABC (with mAb W6/32), heavy chain (mAb HC-10), and *β*_2_m (mAb L368). Isotype-matched Ig serves as the negative control (b).

The differential or concurrent expression of CD44 and CD24 markers has been used for identification of cancer stem cells (CSCs) in a variety of human epithelial [36, 37] and sarcomatoid renal cell carcinoma [38] malignancies. No sHCC CSCs have yet been reported. Therefore, we sought to determine the phenotypic features of the four sHCC cell lines in terms of surface expression of CD44 and CD24. By cytofluorometric analysis, the two HLA class I-negative sHCC29 and sHCC63 cell lines exhibited a CD44^−^/CD24^−^ phenotype, whereas the other two HLA class I-positive sHCC74 and SAR-HCV cell lines showed CD44^+^/CD24^−^ phenotype (Figure 6(a)). These phenotypic differences between the two groups of cell lines were maintained stably, since there was no change in the differential CD44 and CD24 phenotype following the 5th, 13th, and 21st passages and even in cultures recovered from the respective xenografts (data not shown).

### 3.7. Lack of *β*_2_m and HLA Class I Antigen Expression in the Patients' Sarcomatoid HCC Lesions from which the sHCC29 and sHCC63 Cell Lines Originated

To rule out the possibility that the lack of HLA class I antigen expression by cultured sHCC29 and sHCC63 cell lines was caused by *in vitro* artifacts associated with their *in vitro* culture conditions, the surgically removed lesions from which the cell lines were established were analyzed by immunohistochemistry for the expression of *β*_2_m and HLA class I antigens (Figure 6(b)). The *β*_2_m-specific mAb L368, the heavy chain-specific mAb HC-10, and HLA class I-specific mAb W6/32 all stained positive with strong intensity on the cell surface and in the cytoplasm of the tumor lesion from which the HLA class I-positive sHCC74 cell line originated. In contrast, the cell surface HLA class I antigens were not detectable, and yet the cytoplasmic *β*_2_m and HLA class I heavy chains were stained with moderate intensity in the tumor lesions from which the HLA class I-negative sHCC29 and sHCC63 cell lines were derived. The observed immunoreactivity was specific, as the control normal mouse serum or isotype-matched controls stained negative in all tested tumor sections. These findings support the *in vivo* relevance of *β*_2_m loss and HLA class I heavy chain downregulation as detected in the sHCC29 and sHCC63 cell lines *in vitro*.

## 4. Discussion

In this study, we showed that two (sHCC29 and sHCC63) out of the four sHCC cell lines had undetectable surface HLA class I antigen expression which was IFN-*γ*-irreversible. The mechanism underlying this abnormality includes *β*_2_m loss, which is caused by a >49 kb deletion at the *β2M* locus, and marked downregulation of HLA class I heavy chain, TAP1 and LMP7 expression. *β*_2_m is known to be a subunit of HLA class I antigens critical to initiating HLA class I assembly in the endoplasmic reticulum for subsequent membrane transport, while TAP1 and LMP7 are directly or indirectly involved in the formation of an effective peptide-loading platform for HLA class I-*β*_2_m-peptide complex stabilization. To the best of our knowledge, this combination of defects in the HLA class I antigen presentation pathway has never been described in any liver cancer types.

Previous studies have shown that the vast majority of *B2M* gene mutations detected in human malignant tumors are point mutations and microdeletions (a few bases) [39], except for a large (~6 kb) deletion identified in the melanoma cell line FO-1 [40]. Compared to this deletion that spans from the upstream regulatory region to part of exon 2 [40], the >49 kb deletion we identified in two sHCC cell lines extends well across the 15q15 *B2M* locus, which eliminates the entire *B2M* gene. Although we have mapped the 3′ breakpoint to ~22 kb downstream of the gene, we do not know at present how far beyond 27 kb upstream the 5′ breakpoint reaches in each of these two cell lines. In addition, whether this mutation was heterozygous or associated with loss of a wild-type copy remains unclear, although the latter has been reported in the majority of other tumor types with *β*_2_m loss. Furthermore, it is intriguing as to why this *B2M* gene mutation was detected in the two sHCC cell lines that were HBV-associated but not the other two that were HCV-associated. Whether this was a coincidence or a virus-specific phenomenon awaits further analysis of a larger panel of sHCC cell lines.

The combination of *B2M* gene mutation and HLA class I heavy chain or APM downregulation within a single tumor cell population has been described in melanoma [41]. In the present study, we found that both sHCC29 and sHCC63 cells had marked HLA-A, HLA-B, and HLA-C allospecificity, TAP1 and LMP7 downregulation. Notably, only in sHCC63 cells could the downregulated HLA class I heavy chains be slightly upregulated by IFN-*γ*. At variance with these findings, *TAP1* mRNA in sHCC29 cells and *TAP2* mRNA in sHCC63 cells were markedly upregulated, although the basal levels of the tested *TAP1*, *TAP2*, *LMP2*, and *LMP7* mRNA were quite low. Together, these results reflect regulatory defects possibly at different levels. First, *TAP1*, *TAP2*, *LMP2*, and *LMP7* genes may be insufficiently transcribed in sHCC cells in a coordinate fashion, in part because *TAP1* and *LMP2* genes are known to share a bidirectional promoter [42]. Second, some of the downregulated components detected may be under a posttranscriptional control, such as decreased mRNA stability. Third, a defect in the stabilization of multiprotein complex may exist when one or a few of these components are missing or in low quantities.

The two HLA class I-negative sHCC29 and sHCC63 cell lines have more robust *in vitro* growth characteristics and higher xenotransplantability into NOD/SCID mice than the HLA class I-positive sHCC74 cell line. These findings, however, were not associated with a cancer stem cell signature dictated by the CD44^High^/CD24^Low^ phenotype [43] because both sHCC29 and sHCC63 cells were CD44^−^/CD24^−^, whereas sHCC74 and SAR-HCV cells were CD44^+^/CD24^−^. Whether this is a feature unique of sHCC is currently not known. Moreover, the similar growth behavior between the HLA class I-positive SAR-HCV cells and the HLA class I-negative sHCC29 cells suggests that the HLA class I antigen status was independent of basic sHCC biology in the absence of host immunity. The only correlation we have identified thus far is between shorter doubling time *in vitro* and higher xenotransplantability in NOD/SCID mice, although more sHCC cell lines need to be analyzed.

From a therapeutic viewpoint, the lack of HLA class I-*β*_2_m-peptide complex expression at the malignant cell surface will preclude the recognition and destruction by cognate CTLs in immuno-competent hosts [44, 45]. The lack of detection of *β*_2_m in the sHCC tumor lesions from which the two HLA class I-negative cell lines originated indicates that CTLs were unlikely to control these tumor cells in patients [46]. Although not yet confirmed with a large panel of sHCC cell lines and the corresponding lesions because of their rarity, the 50% frequency of HLA class I antigen loss we have revealed in this cancer type strongly suggests a rationale for screening patients for their tumor lesion HLA class I antigen status before treating them with any type of T cell immunotherapy, including immune checkpoint blockade therapy that has recently been shown to be effective in treating some tumor types [18, 19]. With regard to patients with HLA class I-negative sHCC, their treatment options may include mAb-based targeted immunotherapy [47], NK [48] or cytokine-induced killer (CIK) [49] cell-based immunotherapy, and/or chimeric antigen receptor (CAR) T therapy [50]; all of which utilize an alternative approach other than those of the recognition of antigen through T cell receptors to elicit antitumor immunity in the patients.

## Figures and Tables

**Table 1:** Primers used for RT-PCR analysis of *β_2_m* and selected antigen-processing machinery component mRNA expression.

Gene	Nucleotide sequence (5′–3′)	Length (bp)
*B2M*	GGGCATTCCTGAAGCTGACA	424
	TGCGGCATCTTCAAACCTCC	
*LMP2*	TTGTGATGGGTTCTGATTCCCG	449
	CAG AGCAATAGCGTCTGTGG	
*LMP7*	TCGCCTTCAAGTTCCAGCATGG	542
	CAACCATCTTCCTTCAT GTGG	
*TAP1*	TCTCCTCTCTTGGGGAGATG	273
	GAGACATGATGTTACCTGTCTG	
*TAP2*	CTCCTCGTTGCCGGCTTCT	298
	TCAGCTCCCCTGTCTTAGTC	
*GAPDH*	TTCATTGACCTCAACTACAT	469
	GAGGGGCCATCCACAGTCTT	

## Data Availability

The data used to support the findings of this study are available from the corresponding author upon request.

## Supplementary Materials

Supplementary Materials Supplementary Table 1: additional *β*_2_-microglobulin primers used for genomic PCR.
