# Supplementary material for: Total HLA Class I Antigen Loss with the Downregulation of Antigen-Processing Machinery Components in Two Newly Established Sarcomatoid Hepatocellular Carcinoma Cell Lines
Source: J Immunol Res. 2018 Dec 16;2018:8363265. doi: 10.1155/2018/8363265 (PMC6311956; doi:10.1155/2018/8363265)
Supplement: Supplementary Materials — Supplementary Table 1: additional β 2-microglobulin primers used for genomic PCR. [file 8363265.f1.pdf]

Supplementary Table 1. Additional β_2-microglobulin primers used for genomic PCR

Primer name	Sequence 5'~3'	Nucleotide position[a]	Amplicon size (bp)
B2UP-10-F	GATCACTTGAGGTCAGGAGTT	14771-14791	
B2UP-10-R	TCTCTTTATGTTGCCTAGGCT	15103-15123	353
B2UP-9-F	GTTGCCCATTATGTAACAGCT	20105-20125	
B2UP-9-R	GGTTAAAATCTGTTTTGGAACT	20343-20363	259
B2UP-6-F	CCCAGGCTAGAGTGCAGTGGT	22721-22741	
B2UP-6-R	TACAGGCCAGGCATGGTGGCT	22918-22938	218
B2UP-8-F	CTCCAGCCTGGGTGACAGAGT	25002-25022	
B2UP-8-R	GGATCATGGGGGCAGATCCCT	25318-25338	337
B2UP-5-F	CCCTAGGTTTGGGAAGGTCTT	28431-28451	
B2UP-5-R	AGCACTTTGGGAGGCCAAGGT	28764-28784	354
B2UP-4-F	AGTCTTTGCAGTCTGGGCTGT	29603-29623	
B2UP-4-R	CATGGCAGTGCCTCTATGAGT	29855-29875	273
B2UP-1-F	GTGGAGGCGTCTATTCAGCCA	31635-31655	
B2UP-1-R	CCAGCCACTCCACCCAAGGT	32175-32194	560
744-F	CTCTAACCTGGCACTGCGTC	34754-34773	
468-R	TGAG AAGGAAGTCACGGAGC	35018-35037	283
675-F	CCCGATATTCCTCAGGTACTCCAA	38754-38777	
262-R	ACCTCCATGATGCTGCTTAC	39685-39704	951
530-F	TCCACAGGTAGCCTCTAGCAG	41145-41164	
512-R	GGAGTGAGATATAAGAGATAACACAT	41493-41518	376
B2DO-1-F	GGCATGAGCCACTGTGCCCT	43865-43885	
B2DO-1-R	AGCCTCAGGATGGTGCTAGTC	44430-44450	586
B2DO-2-F	TCCAGCCCGGGCAAGAAGAGT	49773-49793	
B2DO-2-R	GAGCCGGAAGTACCTCGGAGT	50061-50081	309
B2DO-3-F	CCAGATACTGGGGTCGTCCTT	55791-55811	
B2DO-3-R	TCTGACCTCCCATCCCGTCAG	56009-56029	239
B2DO-4-F	TTTCAGGGGCCCTTGAATCCT	60620-60640	
B2DO-4-R	CACTCCTTTCCAACCTCTGCCT	60955-60976	357
B2DO-4-2-1-F	TCGACAGCAGCATTCGCGGTT	63427-63447	
B2DO-4-3-R	GTGCTCCTGTATTGGGTGCAT	64159-64179	753
B2DO-4-3-F	ATGCAGAGACACACATAGGCT	63956-63976	
B2DO-4-3-R	GTGCTCCTGTATTGGGTGCAT	64159-64179	224

[a] Designated according to NCBI GenBank Accession AC018901
